# Two Faces of Vitamin C in Hemodialysis Patients: Relation to Oxidative Stress and Inflammation

**DOI:** 10.3390/nu13030791

**Published:** 2021-02-27

**Authors:** Patrick Chaghouri, Nour Maalouf, Sophia Lorina Peters, Piotr Jan Nowak, Katarzyna Peczek, Anna Zasowska-Nowak, Michal Nowicki

**Affiliations:** 1 Student of Medical Faculty, Medical University of Lodz, ul. Pomorska 251, 92-213 Lodz, Poland; patrick_chaghoury@hotmail.com (P.C.); nourmaalouff@gmail.com (N.M.); sophia.peters@stud.umed.lodz.pl (S.L.P.); 2 Department of Nephrology, Hypertension, and Kidney Transplantation, Medical University of Lodz, ul. Pomorska 251, 92-213 Lodz, Poland; katarzyna.peczek90@gmail.com (K.P.); nefro@wp.pl (M.N.); 3 Palliative Medicine Laboratory, Medical University of Lodz, ul. Zeromskiego 113, 90-549 Lodz, Poland; anna.zasowska-nowak@umed.lodz.pl

**Keywords:** hemodialysis, ascorbic acid, concentration, supplementation, oxidative stress, inflammation, antioxidant, prooxidant, cardiovascular risk, oxalate

## Abstract

Hemodialysis (HD) is the most common method of renal replacement therapy. Besides toxins, it eliminates nutrients from the circulation, such as ascorbic acid (AA). HD-patients present AA deficiency more often than representatives of the general population, also due to dietary restrictions. This condition aggravates oxidative stress and inflammation related to uremia and extracorporeal circulation and increases cardiovascular risk followed by mortality. Supplementation of AA seems to be a promising approach in the treatment of hemodialysis patients. Many successful interventions restored plasma AA concentration in HD patients by enteral or intravenous supplementation, concomitantly inhibiting oxidative stress and inflammation. A significant number of studies reported opposite, serious pro-oxidant effects of AA. In this narrative review, we present studies, commenting on their limitations; on AA plasma or serum concentration and the influence of its supplementation on protein and lipid peroxidation, DNA damage, reactive oxygen species generation, paraoxonase activity, advanced glycation endproducts, and C-reactive protein (CRP) concentration. Moreover, in terms of safety, the possible development of oxalosis in HD patients regarding the intravenous or enteral route of AA administration is discussed. Unequivocal clinical results of recent studies on hemodialysis patients are displayed.

## 1. Introduction

In Europe, there are about 140 patients treated with renal replacement therapy (RRT) per million inhabitants. Hemodialysis is the most frequently chosen option of RRT in patients with end-stage chronic kidney disease. Almost 60% of European patients treated with RRT undergo this procedure to sustain their lives. Thirty-seven percent receive a kidney transplant, while only 5% are treated with peritoneal dialysis [1]. During hemodialysis, particles pass from blood through the dialysis membrane according to the concentration gradient, while water is removed from the blood by the created pressure gradient in the dialyzer. Thus uremic, water-soluble toxins are eliminated from circulation and carried away by fluid, but also non-toxic nutrients can be unintentionally eliminated in the same manner, such as ascorbic acid (AA). Hemodialysis patients are affected with increased oxidative stress and inflammation compared to the general population. Therefore, higher amounts of antioxidants, including AA, can be utilized. Their loss and following deficiency are unwanted. Many investigators aimed to answer the question: what is the significance of AA and is there any particular role for it in hemodialysis patients? Furthermore, could this role be modulated with specific nutritional interventions to improve the health condition of this group? In this review, we intend to present studies that tried to establish the extent of AA deficiency among hemodialysis patients, the effectiveness of restoring its proper plasma concentration, the influence on oxidative stress and inflammation, and the safety of AA administration.

## 2. Sources of Inflammation and Oxidative Stress in Hemodialysis Patients

An increase in oxidative stress resulting from the abnormal generation of reactive oxygen species (ROS) is associated with chronic kidney disease [2]. Uremia leads to certain changes of metabolism on the intracellular and organ level. Mitochondrial dysfunction with a remarkable decrease of cytochrome C oxidase activity and an increase of nicotinamide adenine dinucleotide (NADH) expression was found in mononuclear cells from the peripheral blood of end-stage chronic kidney disease patients regardless of hemodialysis [2,3]. The consequent abnormal mitochondrial transmembrane potential leads to overproduction of ROS in the respiratory chain [4], mainly superoxide anion radicals, which damage mitochondria and leak out and damage surrounding cellular structures [3]. The highly reactive form of oxygen can be converted by superoxide dismutase to oxygen and hydrogen peroxide (H_2_O_2_). H_2_O_2_ is less active than superoxide but still highly reactive; therefore, it can further react with other compounds, oxidizing and damaging them. 

**Figure 1:**
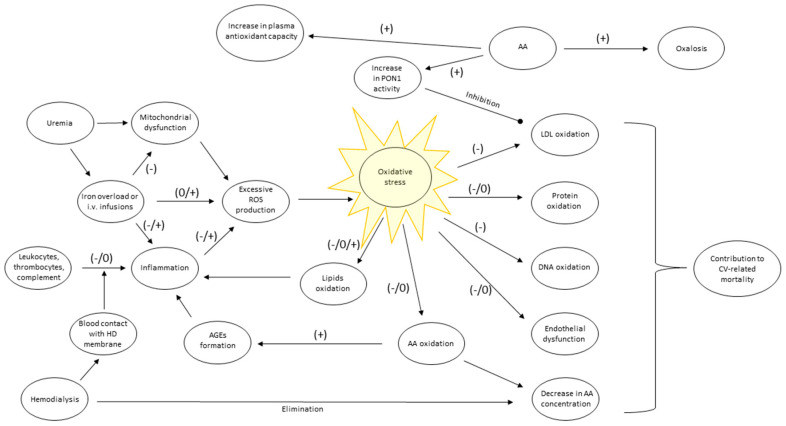
Primary sources and consequences of oxidative stress in hemodialysis patients, and influence of AA administration on oxidative stress-related processes reported in studies on hemodialysis patients, presented later: (-) inhibition, (0) no effect, (+) augmentation of a process by AA administration. AA, ascorbic acid; AGEs, advanced glycation end-products; CV, cardiovascular; HD, hemodialysis; LDL, low-density lipoprotein; PON, paraoxonase; ROS, reactive oxygen species.

The abundance of mitochondria in a human organism, together with their permanent and improper activity, can be a significant ROS source in uremic patients. Initiation of hemodialysis reduces the level of uremia but introduces the new pro-inflammatory factor, which is a repeated contact of a patient’s blood with artificial material [5]. A dialyzer contains multiple capillaries of total surface approximating 2 m^2^. During the session, the patient’s blood is pumped to extracorporeal circulation to enter dialyzer capillaries made of a certain dialysis membrane type. Different types of membranes are characterized by their biocompatibility. Lower biocompatibility of a membrane (e.g., made of cuprofan) means stronger stimulation of immune-competent cells, while membranes of high biocompatibility (e.g., polysulfone) stimulate those cells weakly [2,6,7]. Capillaries are submerged in dialysis fluid, which flows around them in the opposite direction than blood does. Trace amounts of immunogenic lipopolysaccharide (LPS) may be present in the fluid and contribute to inflammation initiation if LPS concentration in dialysis fluid is not properly controlled [2]. In most cases, hemodialysis is performed three times a week and lasts approximately 4 h each. Prolonged and repeated contact of leukocytes, thrombocytes, and plasma with hemodialysis membrane, which acts as a foreign body, stimulates immune response and inflammation [8]. Activated leukocytes release cytokines, undergo degranulation, releasing ROS, mainly H_2_O_2_ [2,6,7]. Moreover, contact with artificial membrane activates platelets, complement, and clotting factors, which intensifies inflammation. Therefore, the higher the dose of dialysis, the stronger inflammation and oxidative stress can occur [2]. The serum concentration of basic inflammatory marker C-reactive protein (CRP) is typically higher among hemodialysis patients than in the general population [9]. Treatment of anemia with intravenous iron leads to ROS generation, too [2]. Thus, besides uremia, RRT is a source of ROS itself (Figure 1).

Regardless of ROS’s origin, their high chemical activity makes them react intensively with various cellular structures, leading to their damage. For instance, oxidative changes in lipids of erythrocytes’ membranes lead to their higher susceptibility to hemolysis and aggravation of anemia [10]. The intensity of oxidative damage can be measured with the following specific markers, which are products of oxidation of the following structures: (1) lipid membranes: thiobarbituric acid reactive substances (TBARS, react with thiobarbituric acid, which is used for their detection), malondialdehyde (MDA), phosphatidylcholine hydroperoxide, isoprostanes; (2) lipoproteins: oxidized LDL; (3) proteins: advanced oxidation protein products (AOPP), protein carbonyls; (4) DNA: 8-hydroxy-2’-deoxyguanosine (8-OHdG) [2,8]. Oxidized LDL is proatherogenic [11], AOPP indicates albumin oxidation [12], 8-OHdG is a mutagenic product of 2′-deoxyguanosine oxidation [13]. ROS impair the endothelium function, decreasing its reactivity and signal transduction, resulting in blood flow limitation and contribution to atherogenesis [14]. Increased oxidative stress associated with uremia and renal replacement therapy is one of the factors precipitating cardiovascular diseases (Figure 1). Those diseases are the leading cause of death in this group of patients [8,15]. Human plasma contains various substances that can react with ROS to compete with mentioned compounds for being oxidized. This ability can be measured with specific experimental systems such as ferric reducing ability power (FRAP) or with the ratio of concentrations of known antioxidants to their oxidized forms, for instance, glutathione/glutathione disulfide (GSH/GSSG) or ascorbate/dehydroascorbate [16,17,18]. Concentration or expression of specific enzymes involved in ROS degradation, such as Cu/Zn superoxide dismutase, can also be quantified [19]. On the other hand, ROS production by immune cells can be easily identified in systems containing luminescent or fluorescent probes (e.g., lucigenin, dichlorofluorescein), which emit a certain length of a light wave on oxidation [20,21]. The aspects mentioned above of oxidative stress concerning vitamin C and hemodialysis patients will be presented in the following chapters. 

## 3. Vitamin C potential Against Inflammation and Oxidative Stress

Ascorbic acid is a water-soluble compound with a molar mass of 176 Da, which, after absorption in the intestines, penetrates the extracellular and intracellular compartments of the human body [22,23]. Its chemical structure, consisting of hydroxyl groups attached to a furan ring, makes it a relatively easy donor of electrons and protons. For this reason, it can take part in redox reactions. AA can be oxidized simultaneously, reducing other ROS compounds, especially superoxide anion radical [24]. Competitive reactions of AA with ROS scavenge them before they reach various cell components. Thus, AA is oxidized instead of lipid membranes, proteins, and DNA, protecting these structures from being damaged [25]. Oxidation transforms AA to dehydroascorbic acid, which can be enzymatically converted back to AA using glutathione [18,24,25]. In this reaction, GSH is enzymatically oxidized to GSSG by trans-dehydrogenases, while dehydroascorbic acid is reduced to AA. The cost is a decrease in GSH concentration and GSH/GSSG ratio. Therefore, supplementation of AA to GSH-deficient rats reduced their dependence on GSH as it did not have to be utilized for AA regeneration [18]. 

Vitamin C in vitro not only scavenged ROS but also inhibited their formation. The suppressor effect on Jak2/Stat1/IRF1 signaling pathway in the endothelium reduced the production of superoxide radicals, hydrogen peroxide, and peroxynitrite, a powerful oxidant and nitrating agent [26]. AA inhibited inducible nitric oxide synthase, preventing excessive production of nitric oxide, a substrate for peroxynitrite generation in the presence of superoxide anion radicals [26]. In vitro studies bring uncertainty regarding antioxidative features of AA as some reported its prooxidative actions. AA’s presence caused highly reactive hydroxyl radicals generation in enzymatic or chemical systems containing copper or iron [27,28].

AA is accumulated in leukocytes where it can reach concentrations 50- to 100-fold higher than in plasma, which potentiates possible anti-inflammatory effects [25]. For instance, peripheral blood lymphocytes incubated with AA and then stimulated with LPS generated smaller amounts of tumor necrosis factor (TNF)-α and interferon-γ (pro-inflammatory cytokines) and more significant amounts of the anti-inflammatory interleukin (IL)-10 [29]. AA inhibited the synthesis of the pro-inflammatory cytokines TNF-α and IL-6 in monocytes isolated from patients with pneumonia [30]. It inhibited TNF-α-induced adhesion molecule expression on endothelial cells, necessary for leukocyte stickiness and sludging [26]. AA inhibited NF-κB, a pro-inflammatory transcription factor activation in vitro in human epithelial cell line (ECV304) and human umbilical vein endothelial cells culture (HUVECs) [31] and suppressed netosis of granulocytes [32]. On the other hand, AA presented pro-inflammatory features stimulating granulocyte chemotaxis, lymphocyte proliferation, interferon production, and increasing antibody blood concentration [33]. AA moreover enhanced fibroblast proliferation and migration, phagocytosis, and ROS generation by granulocytes [32]. It increased hydroxyl radical production by activated human leukocytes and murine macrophages in vitro [27]. 

Regarding the antioxidative and anti-inflammatory properties of AA, it was reasonable to test the influence of this vitamin on hemodialysis patients with the hope to alleviate chronic oxidative stress and inflammation, which is more intense than among healthy subjects. However, if we consider the pro-oxidant and pro-inflammatory features of AA, the results can be surprising.

## 4. Concentration of Ascorbic Acid in Plasma and Serum of Hemodialysis Patients

Vitamin C deficiency is an essential factor to take into consideration in patients on maintenance hemodialysis. Deicher et al. prospectively over 30 months studied the association of AA concentration in plasma with the frequency of cardiovascular events in 138 patients on maintenance hemodialysis and hemodiafiltration. AA plasma levels were recorded before the dialysis session. Hemodiafiltration’s technique comprises removing many water liters from patients’ blood during an otherwise hemodialysis session and replacing it with a substitution fluid. All patients were observed for myocardial infarction, aortocoronary bypass grafting, stroke, limb amputation because of peripheral vascular disease and cardiovascular mortality, and all causes of mortality. The results showed that patients with AA concentration in plasma lower than 32 μmol/L were at almost a fourfold higher risk for major cardiovascular events and cardiovascular mortality compared to patients with AA concentration in plasma exceeding 60 μmol/L [15]. Deicher et al. had a relatively large study group aiding in the strength of their study. Similar observations of an association between low concentration of AA in plasma and shorter time to death were made by Dashti-Khavidaki et al. on 91 maintenance hemodialysis patients [34]. These studies suggest that AA can be considered a factor in preventing fatal and non-fatal major cardiovascular events in hemodialysis patients. Therefore, AA can be recognized as a gateway antioxidant in hemodialysis patients, where low concentrations in plasma can be harmful. 

Plasma levels of AA in hemodialysis patients were addressed in several reports. Papastephanidis et al. (Table 1) presented that hemodialysis patients compared with healthy subjects have a fourfold smaller mean of AA concentration in serum collected before the dialysis procedure, which was 12 μmol/L [35]. It could be explained by decreased AA dietary intake and clearance of AA during hemodialysis. A low-potassium diet, advised in end-stage chronic kidney disease, limiting fruits and vegetable consumption may be accompanied by decreased vitamin C content. The low molecular mass of ascorbic acid, which is comparable to the molecular mass of creatinine (113 Da), facilitates its quick diffusion across dialysis membrane and elimination with dialysate [35,36]. However, this early study utilized the enzymatic AA determination method in serum, which had limited precision. Canavese et al. (Table 2), in a more recent study, utilized the high-performance liquid chromatography (HPLC) method of AA determination in plasma, obtaining similar results based on 18 hemodialysis patients. Although the AA determination method was accurate, the studied group was small, and the inclusion criterion was AA deficiency, defined as serum AA concentration < 15 μmol/L. The mean AA concentration was 9 μmol/L [37]. The same study proved that hemodialysis eliminates AA from plasma, reducing its concentration by 38 ± 23% to 59 ± 12% with a parallel appearance of this compound in the dialysate [37]. Washio et al. also demonstrated on 16 patients that hemodialysis sessions in which their doses, unfortunately, were not disclosed in the study, decreased the mean concentration of AA in plasma by 67% from 43.6 to 14.6 μmol/L [19]. The authors provided neither the length of the dialysis session nor other possible parameters describing the dialysis dose makes their finding hardly applicable in clinical considerations [19]. Eiselt et al., who examined 20 patients dialyzed with Kt/V = 1.44, described a 62% decrease in AA serum concentration during a hemodialysis session [38]. 

Besides dialysis-related elimination, the role of diet and intake of AA can be meaningful in maintenance hemodialysis patients. Lim et al. studied their nutritional status with the duration of dialysis. Dietary management in the end-stage renal disease populations is necessary to maintain adequate caloric intake and prevent hyperphosphatemia and other electrolyte imbalances. In this cross-sectional study, the population (*n* = 145) was divided into groups according to hemodialysis vintage; group 1 less than one year, group 2 between 1–5 years, and group 3 more than five years on hemodialysis. AA intake was determined based on informed questionnaires. The study showed that AA intake in group 3 was 20% lower than in group 1 [39]. The findings highlighted the importance of vitamin C content in hemodialysis patients’ diet, decreasing with the treatment duration. These situations may have noxious consequences for patients’ health [39]. Besides the risk for cardiovascular diseases, the final complication, scurvy, is the extreme clinical outcome of severe and chronic vitamin C deficiency, which was occasionally reported in dialysis patients [40,41]. Therefore, AA supplementation can be beneficial, especially in specific subpopulations of hemodialysis patients [39]. 

Deicher et al. conducted a cross-sectional study on hemodialysis and hemodiafiltration patients investigating plasma vitamin C levels with the HPLC method (Table 1). As in Papastephanidis’s and Canavese’s studies, the blood samples were taken before the dialysis session, but the AA concentration in plasma was at least 4-fold higher than those reported earlier [42]. Moreover, the measured median concentration of 45 μmol/L was similar to mean concentrations measured in the general population, which was approximately 8–10.5 mg/L (45–60 μmol/L) [43,44]. The mean concentration measured in the plasma of hemodialysis patients by Richter et al. was in the same range (59 μmol/L), and each study had more than a hundred participants; however, hemodialysis dose remained unknown in the latter [45]. This finding could undermine an earlier statement about AA deficiency in hemodialysis patients; however, if we look at the distribution of AA concentration among studied populations, specific differences between hemodialysis patients and healthy subjects can be observed. Three studies conducted by Richter et al., Raimann et al., and Zhang et al. (Table 1), analyzed the distribution of AA levels in hemodialysis patients’ plasma. Raimann conducted his study on a small group, while studies of the other authors have a relatively high number of participants regarding research on hemodialysis patients. They established that 15%, 25%, and 38% of those patients, respectively, have the AA concentration in plasma collected before hemodialysis lower than 10–11 μmol/L [45,46,47].

In contrast, in a representative group of 897 people derived from Belgium’s general population, only 6% had an ascorbic acid concentration in plasma lower than 20 μmol/L. Belgian participants were not taking AA supplements [43]. In 300 German volunteers, 13% presented the plasma concentration below 28 μmol/L and only 3% below 8.5 μmol/L. People taking AA supplements were not excluded from this study [44]. Considering this data, it becomes apparent that although mean AA concentration in the plasma of hemodialysis patients and representatives of the general population is similar, low plasma concentrations occur more frequently in hemodialysis patients than in the general population.

Moreover, hemodialysis patients in mentioned studies were allowed to take vitamin supplements independently as they had been doing before the studies; therefore, the prevalence of low AA concentration in plasma could be higher if patients taking supplements were excluded. On the other hand, if mean concentrations of AA are similar in healthy subjects and hemodialysis patients, while the incidence of low concentration is higher among the latter, simple mathematics tells us that abnormally elevated concentrations of AA have to be encountered in hemodialysis patients more frequently than in the general population, as well. Possibly, those concentrations can precipitate unwanted effects of uncontrolled AA supplementation, discussed later. 

An investigation on the impact of hemodialysis on AA concentration in serum was undertaken again by Coveney et al. (Table 1), who noted that patients hemodialyzed for over 15 h per week are more likely to have water-soluble vitamin deficiency compared to patients who had shorter dialysis time. Before the hemodialysis session, the extended time group had a lower median AA concentration than the conventional group [48]. The doubled hours of dialysis in the extended hemodialysis group, meaning elongation of the time where AA passed from the cellular compartment to plasma and from there through dialysis membrane to dialysis fluid, led to these expected results. Coveney’s approach to conventional and extended hemodialysis created a critical consideration when debating on whether to give AA supplementation to hemodialysis patients or not. As the extended hours of dialysis became more common among RRT techniques, the evaluation of AA levels and AA’s supply become more reasonable because of the higher possibility of AA deficiency. However, the study had a weakness. The Kt/V ratio, an indicator of dialysis dose and adequacy describing its effectiveness, was not provided [48].

Broader comparisons with other studies and generalized conclusions were, in turn, difficult. Raimann et al. (Table 1) partially undermined the relationship between the dose of dialysis and the AA concentration in plasma, comparing the effect of two different dialysis frequencies on the AA plasma levels. Patients were participants of the Frequent Hemodialysis Network (FHN) trial and were divided into two groups; conventional and frequent hemodialysis consisting of 3 and 6 hemodialysis sessions per week, respectively. Each frequent session duration was 1/3 shorter than conventional but had almost the same Kt/V ratio (per session), which means the sessions had to be more intense. Both groups had similar oral AA supplementation during the study’s whole duration, starting from the baseline point. This study showed no difference in the mean AA concentration between the two groups, although the total dose of dialysis measured with weekly Kt/V was much higher in the frequent group [47]. Although limited by the small number of study participants (*n* = 20), this observation may suggest that elimination of AA during hemodialysis is insignificant. Although, if we consider these results with Coveney et al., the other conclusion seems plausible. The hemodialysis session’s duration has a more substantial impact on AA elimination than its dose measured with Kt/V, a urea elimination-based parameter. Since AA molecular weight is 3-fold higher than urea’s, and it is abundant in the intracellular compartment [22,23,37,49], it possibly diffuses slowly across cellular membranes to the blood before subsequent elimination in the dialyzer and binds to albumin, which holds it in the plasma [36]. Therefore, its kinetics of elimination differs from urea [37]. Thus, it made dialysis session’s length a key factor for its elimination rather than an initial high concentration gradient across the dialysis membrane. Moreover, such kinetics contributes to relatively mild AA elimination from both intracellular and extracellular compartments together during hemodialysis sessions [37]. However, the decrease in plasma AA concentration during hemodialysis is remarkable; plasma accounts only for 10% of total body fluids. Single hemodialysis with Kt/V aimed > 1.2 eliminated 38 mg of AA in patients with low plasma levels and 100 mg in patients with restored normal levels after successful AA supplementation [37]. We can collate it with highly changeable AA intake in hemodialysis patients’ diets, ranging from 40 to 300 mg/day, to find a possible explanation for heterogeneity of presented results of AA concentration in blood [50]. Similar to cross-sectional studies presented in Table 1, interventional studies on small groups where baseline AA concentration was measured display high variability (Table 2) with the mean value ranging from 9 to 44 μmol/L. 

An additional factor responsible for differences in the results of AA concentration obtained by researchers is the method of AA determination and the initial preparation of plasma samples. This issue was postulated by Kennedy et al., who found that plasma stabilization with metaphosphoric acid prevents AA’s remarkable spontaneous degradation. When metaphosphoric acid was applied, the AA’s concentration in the same patient was 40% higher [51]. Approximately half of the analyzed studies (marked in Table 1 and Table 2) utilized metaphosphoric acid in laboratory preparations while the others did not. 

In conclusion, the studies mentioned above show that hemodialysis decreases AA concentration in patients’ plasma, but this influence is limited, changeable, and depends on the dialysis setting. The most influential variable is the length of the dialysis session. Dietary restrictions contribute to decreased intake of AA. Those factors resulted in AA deficiency among hemodialysis patients, which is associated with increased morbidity and mortality. Certain studies reported low mean or median AA levels, suggesting widespread AA deficiency. The others reported a higher incidence of abnormally low AA levels among hemodialysis patients, while the mean or median concentration in plasma was close to the one measured in the general population. Differences in plasma preparation and AA determination methods, local dietary habits, allowance for vitamin supplements intake, together with the limited number of participants, can be partially responsible for observed discrepancies. Mentioned findings suggest that intervention is needed in hemodialysis patients to maintain AA levels at their normal ranges and avoid its deficiency’s complications. However, not all individuals on hemodialysis require it.

**Table 1:** Studies on ascorbic acid concentration in serum or plasma of hemodialysis patients.

Dialysis Modality (Study’s Country)	Study Description	Main Results	References
Hemodialysis (Greece)	Cross-sectional, 93 HD patients (aged 19–71 years, HD dose 12 h/week, HD vintage 10–137 months), 52 healthy controls, AA determination with the enzymatic method in pre-HD serum.	HD patients presented 4-fold smaller mean AA concentration than healthy subjects, 12.0 ± 8.1 vs. 50.0 ± 22.1 μmol/L respectively.	Papastephanidis et al.(1987) [35]
Hemodiafiltration or hemodialysis (Austria)	Cross-sectional, 130 HD/HDF patients (median: age 60, 74% on HDF, HD/HDF vintage 20 months, Kt/V 1.39). AA determination with the HPLC method in pre-HD plasma.	Median AA concentration was 45.1 μmol/L, IQR: 24.3–76.1 μmol/L.	Deicher et al.(2004) [42]
Hemodialysis (NYC, USA)	Cross-sectional, 117 HD patients (mean: age 63, HD vintage 3.2 years, HD dose unknown). AA determination with the HPLC in pre-HD plasma ^m^	Mean AA concentration was 58.9 ± 65.3 μmol/L. Values < 10 μmol/L, between 10 and 80 μmol/L and > 80 μmol/L observed in 15%, 66% and 19% percent of patients respectively.	Richter et al.(2008) [45]
Hemodialysis (China)	Cross-sectional, 117 HD patients (mean: age 59, HD vintage 49 months, Kt/V 1.6) AA determination with the HPLC in pre-HD plasma ^m^.	AA concentration < 2 μg/mL **, between 2- 4 μg/mL and > 4 μg/mL was present in: 38%, 27% and 35% of patients respectively	Zhang et al. (2011) [46]
Conventional and extended hours hemodialysis (Australia)	Cross-sectional, 26 patients in EHD (mean age: 55, HD vintage 25.7 months, HD dose 28 h/week) 26 in CHD (mean: age 71, HD vintage 57 months, HD dose 13.5 h/week). AA determination with HPLC in pre-HD serum.	Median pre-HD AA concentration was 3.8-fold lower in the EHD than the CHD group (0.30 vs. 1.14 mg/dL *).	Coveney et al.(2011) [48]
Conventional and frequent hemodialysis (USA)	Participants of the FHN trial, NCT00264758. CHD group: 20 patients, mean age 50.6, 3 HD sessions per week, median duration 3.5 h, Kt/V = 1.46. FHD group: 24 patients, mean age 48.8, 6 HD sessions per week, median duration 2.4 h, Kt/V = 1.43. AA determination with the HPLC in pre-HD plasma ^m^.	Mean AA concentration approximated 25 ± 22 μmol/L and did not differ between groups and time points. 25% of patients have AA concentration < 10 μmol/L.	Raimann et al. (2019) [47]

* Conversion factor for units: vitamin C mg/dL to μmol/L × 56.78. ** Conversion factor for units: vitamin C μg/mL to μmol/L × 5.678. ^m^ Plasma stabilization with metaphosphoric acid. AA, Ascorbic Acid; CHD, Conventional hemodialysis; EHD, extended hemodialysis; FHD, frequent hemodialysis; HD, Hemodialysis; HDF, Hemodiafiltration; HPLC, High-performance liquid chromatography; IQR, interquartile range; Kt/V, parameter of dialysis dose.

## 5. Influence of Ascorbic Acid Supplementation on Its Concentration in Serum and Plasma of Hemodialysis Patients 

Since ascorbic acid deficiency is more frequent in hemodialysis patients than in healthy subjects, as discussed earlier, researchers started studying the effect of AA supplementation on its plasma concentration to find a dose useful in maintaining the concentration within normal ranges. The recommended daily intake of AA for healthy adults is 75 mg and 90 mg for females and males, respectively [52]. Another approach was needed to treat AA deficiency, and an investigation of the effect of higher doses was undertaken. De Vriese et al. (Table 2) studied the effect of 360 mg and 1500 mg per week of oral AA supplementation on pre-dialysis AA serum levels, determined with the spectrophotometric method, which is less precise than HPLC used by other researchers. 120 mg of AA was given after each dialysis session then followed by a dose of 500 mg. The authors observed an increase in the mean serum AA concentration. Nonetheless, 360 mg per week, a dose smaller than the one advised in healthy people, was not enough to reinstate AA status in this group since 26.5% of patients sustained serum AA levels below the minimum reference limit of 0.2 mg/dL (11 μmol/L). Following the dosage of 1500 mg per week, there was a shift in the patient population, and the majority came into the reference limits of serum AA levels, with 6.7% remaining below [53]. De Vriese et al. experimented with AA dosage, making it clear that the dose higher than recommended for supplementation in healthy people effectively increased AA serum levels in hemodialysis patients. No adverse effects from the intervention group were reported [53].

In another observational study conducted by Washio et al. (Table 2), oral AA supplementation of 200 mg, 400 mg, and 1000 mg was given each month, thrice weekly for three months. The study showed an increase in mean AA concentration in pre-hemodialysis plasma, measured with the HPLC. AA concentration increased with the additional dosage of AA supplementation, reaching a maximum mean of 138.2 ± 51.5 μmol/L, which means the complete resolution of AA deficiency and even exceeding the upper reference limit [19]. In contrast to De Vriese’s experiment, the effect of supplementation was visible even with the initial dose, which weekly equaled a dose advised in healthy people. These findings are most probably a result of the different construction of both studies. De Vriese assessed AA concentration in plasma approximately 44 h after AA administration, while Washio only 1 h after the ingestion [19,53]. The former investigated steady concentration in its stable phase while the latter peak concentration in the absorption phase [22]. The other limitation of the latter study was also a much smaller number of 16 patients. That is why De Vriese et al., although not utilizing the HPLC method, bring more reliable results than Washio’s.

In contrast with Washio, Zhang et al. selected a relatively large study group of 100 participants, intending to create a more reliable randomized cross-over trial on patients with a low initial AA concentration (baseline concentration in plasma < 23 μmol/L, mean 8.5 ± 4.5 μmol/L; Table 2). Unfortunately, information about the time-relation between ingestion of ascorbic acid and its concentration measurement is missing, which largely limits the reliability of obtained results because AA concentration in plasma becomes stable not earlier than 4 h after ingestion [22]. 200 mg/day of oral AA supplementation was given for three months alternatively with no intervention. The results showed an increase in pre-hemodialysis plasma AA levels in most patients, reaching a mean of 59 ± 58.5 μmol/L [54]. Candan et al. proved ingestion of 250 mg of AA a day increased its plasma concentration by 1/3 (Table 2). 

Nonetheless, the study has certain drawbacks; the authors did not use metaphosphoric acid to stop AA degradation, applied fluorimetric method of determination instead of HPLC, and did not reveal hemodialysis vintage of the studied population making its representativeness uncertain [10]. An oral dose of 250 mg thrice weekly was effective in Fumeron and Chan et al.’s studies (Table 2) and increased AA level to normal ranges [17,55]. Eiselt et al. elevated AA’s concentration in the serum of 20 patients from 16 to 60 μmol/L with an initial dose of 250 mg/day for two weeks followed by a maintenance dose of 50 mg daily given for the same time [38]. This way, the authors combined the effectiveness of higher doses needed for effective AA supplementation with the safety of smaller doses, which maintained AA concentration improvement (measured with HPLC method after plasma stabilization with metaphosphoric acid). In response to interventions, AA deficiency in hemodialysis patients ameliorates. None of the mentioned studies reported the occurrence of unwanted clinical effects of AA supplementation.

Hemodialysis patients have dialysis access through a catheter installed to the vena cava, or an arterio-venous fistula created in a limb. This ensures, besides dialysis procedure, easy access for intravenous administration of medications. Therefore, this route of AA administration has been examined as well. A small, longitudinal, observational study conducted by Canavese et al. (Table 2) proved the effectiveness of 500 mg i.v. of AA a week in reversing its deficiency [37]. 300 mg of AA infused thrice weekly after hemodialysis sessions doubled its plasma level after eight weeks of observation by Tarng et al. [21]. Both studies utilized metaphosphoric acid for sample stabilization, while the former applied HPLC determination of AA and the latter colorimetric method (Table 2). Comparing the administration of the same dose of AA in both ways performed by Chan et al. (Table 2) indicated intravenous route as more effective against AA deficiency [55].

In a more extensive, placebo-controlled observational study conducted by El Mashad et al. (Table 2), the authors investigated the effect of intravenous AA supplementation on children’s serum AA levels. Intravenous administration provides better compliance, especially in this group of patients. 250 mg of AA was injected thrice weekly for 12 weeks. Pre-dialysis serum levels of AA more than doubled in the supplemented group while remained unchanged after placebo. The experiment was free of unwanted effects [56]. El Mashad et al. observed the pediatric population, focusing on a less popular choice of patients, advances our understanding of whom AA supplementation benefits. 

In conclusion, the results of mentioned studies show that both oral and intravenous AA supplementation can increase the plasma AA concentration in hemodialysis patients, thus preventing AA deficiency occurrence without remarkable side effects. Enteral doses of 750 mg per week, approximately 20% higher than AA intake advised in healthy people, restored proper AA concentration in plasma. Intravenous doses of 500–900 mg/week were effective; however, analysis of potential unwanted effects of such intervention related to oxalate formation, conducted by Canavese et al., holds this therapeutic approach questionable [37]. The issue of safety is discussed in the ninth chapter of this review. 

**Table 2:** Influence of ascorbic acid supplementation on its concentration in serum and plasma of hemodialysis patients.

Objective (Study’s Country)	Study Description	Main Results	References
Evaluation of the effects of oral AA supplementation on AA concentration in plasma. (Turkey)	Prospective, open label, randomized, placebo-controlled trial, 34 HD patients (mean age 46, HD vintage unknown, dose 12 h/week) received orally 250 mg of AA/day for 90 days (*n* = 15) or placebo. The concentration of AA in pre-HD plasma was measured with fluorimetry.	AA concentration increased from 32 ± 13 to 46 ± 16 μmol/L, remaining unchanged after placebo.	Candan et al. (2002) [10]
Evaluation of the influence of intravenous AA supplementation on AA concentration in plasma. (Taiwan)	Prospective, open label, randomized, placebo-controlled trial, 51 HD patients (mean: age 59, HD vintage 46 months, HD dose 12–13.5 h/week) 26 in the placebo group, 300 mg of AA or saline was infused after each HD for 8 weeks, pre-HD AA in plasma ^m^ was measured with colorimetric method.	AA concentration doubled from initial 44 ± 19 μmol/L, remaining unchanged after placebo.	Tarng et al. (2004) [21]
Assessment of the effects of intravenous AA supplementation on AA concentration in plasma. (Italy)	Prospective observational study; 14 HD and 4 HDF patients with AA deficiency (mean: HD vintage 9.9 years, Kt/V ≥ 1.2) AA was infused once a week after HD in a dose of 250 mg for 3 months and 500 mg for the following year. AA determination with HPLC in pre-HD plasma ^m^ samples.	Mean concentration of AA raised from initial 1.6 ± 0.8 mg/L ^#^ to maximal 6.6 ± 2.8 mg/L.	Canavese et al. (2005) [39]
Evaluation of the effects of intravenous or enteral AA supplementation on AA concentration in plasma. (Australia)	Prospective, randomized, parallel observational study, 21 HD patients (mean: age 56, HD vintage > 6 months, sessions thrice a week, URR 76%) received 250 mg of AA orally or IV after HD sessions for 8 weeks. AA measurements with HPLC in pre-HD plasma.	Mean concentration of AA increased 2.8 fold from initial 2.8 ± 0.7 mg/L ^#^ in the enteral group and 3.4 fold from initial 1.8 ± 0.5 mg/L ^#^ in IV group.	Chan et al. (2005) [55]
Assessment of the effect of oral AA supplementation on AA concentration in plasma. (France)	Prospective, randomized, observational study, 33 HD patients (mean: age 52, HD vintage 6.1 years, session duration 4,2 h thrice a week, Kt/V 1.2) received 250 mg of AA orally after each HD for 2 months or no drug. AA in pre-HD plasma was determined with HPLC.	Mean AA-concentration increased 3.4 fold from the initial 19.5 ± 13.5 μmol/L in the supplemented group only.	Fumeron et al. (2005) [17]
Studying the effect of oral AA supplementation on AA concentration in serum. (Belgium)	Prospective observational, study. 92 HD patients (mean: age 67, HD vintage 2.9 years, Kt/V 1.4) AA was administered orally, for 3 months, after HD sessions at a dose of 360 or 1500 mg/week. Pre-HD AA concentration was determined with the enzymatic spectrophotometry.	Median AA concentration increased from 0.22 to 0.33 and to 0.63 mg/dl* after supplementation of 360 and 1500 mg/week, respectively.	De Vriese et al. (2007) [53]
Studying the effect of oral AA supplementation on concentration of AA in plasma. (Japan)	Prospective, observational study, 16 HD patients (mean: age 64, HD vintage 7.9 years, HD dose unknown). 1st month of the study: oral administration of 200 mg of AA 1 h before HD, thrice a week. 400 mg and 1000 mg were given during the 2nd and the 3rd month, respectively. AA in pre-HD plasma ^m^ was determined with HPLC.	Baseline mean AA concentration was 43.6 μmol/L and increased 2.1, 2.8 and 3.2 folds after consecutive doses of 200, 400, and 1000 mg respectively.	Washio et al.(2008) [19]
Studying the effect of oral AA supplementation on HD-patients with initially low AA level in plasma. (China)	Prospective, randomized, cross-over, observational study, 100 AA deficient HD patients (mean: age 64, HD vintage 48 months, Kt/V 1.5, initial AA concentration in plasma < 4 μg/mL *) received 200 mg/day of AA orally or no drug, for 3 months. AA was determined with HPLC in pre-HD plasma ^m^.	AA concentration raised above 4 μg/mL ** in 80% of patients exceeding baseline value 4.5 to 7 folds.	Zhang et al. (2013) [54]
Studying the effects of intravenous AA supplementation on AA concentration in serum of children treated with HD. (Egypt)	Prospective, open label, randomized, placebo-controlled trial, 60 children (mean: age 9 years, HD vintage 2.9 years; HD dose 9–12 h/week, Kt/V ≥ 1.2) 30 patients received 250 mg of AA IV after each HD session for 12 weeks, or placebo. AA determination in pre-HD serum with HPLC.	Mean AA concentration increased 2.5 folds from initial 8.97 ± 4.38 μmol/L. It remained unchanged after placebo.	El Mashad et al.(2016) [56]

* Conversion factor for units: vitamin C mg/dL to μmol/L × 56.78. ** Conversion factor for units: vitamin C μg/mL to μmol/L × 5.678. # Conversion factor for units: vitamin C mg/L to μmol/L × 5.678. ^m^ Plasma stabilization with metaphosphoric acid. AA, Ascorbic Acid; HD, Hemodialysis; HDF, Hemodiafiltration; HPLC, High-performance liquid chromatography; IV, Intravenous; Kt/V, parameter of dialysis dose; URR, urea reduction ratio.

## 6. Inhibitory Effects of Ascorbic Acid Supplementation on the Intensity of Oxidative Stress among Hemodialysis Patients

Vitamin C can act as an antioxidant and free-radical scavenger that protects cellular structures from oxidative damage by being oxidized itself. This hypothesis is supported by the observation of Washio et al. (Table 2), highlighting that the concentration of the oxidized form of AA did not change during the hemodialysis session. In contrast, the reduced form decreased, resulting in increased oxidized AA to native AA ratio. This phenomenon was evident in patients receiving oral AA supplementation of 1000 mg thrice a week, who had the AA concentration in plasma elevated. In this case, the hemodialysis session triggered a substantial decrease of native AA concentration and a significant increase in the concentration of oxidized AA, and higher growth of oxidized AA to native AA ratio [19]. Such oxidation of AA concurrent to lipid oxidation, for example, would inhibit reactive oxygen species (ROS)-related cellular damage. Washio et al. study was limited by a small number of participants (*n* = 16) and lack of information about hemodialysis dose and membrane, which would enable the assessment of exposure to particular artificial material of specific biocompatibility and ability to stimulate inflammation and oxidative stress [6,7].

Yang et al. observed (Table 3) that intravenous AA administration during dialysis sessions had a strong antioxidant effect inhibiting ROS formation, mainly H_2_O_2_ in blood. Lipid peroxidation, measured with the concentration of phosphatidylcholine hydroperoxide in plasma, was inhibited. Those two parameters showed a sharp decrease compared to the control group, which underwent no intervention. Furthermore, the total antioxidant status of patients’ plasma significantly improved compared to controls [57]. A placebo was not applied in this study, and the total number of participants was not high (*n* = 20); while the study’s strengths include monitoring of AA concentration in plasma, which proved its rise after AA infusion and a decrease related to hemodialysis. These findings are convincing in attributing observed protection of lipids to AA. 

Another lipid-protecting action of AA was demonstrated by Ferretti et al. (Table 3), who studied the effect of intravenous AA supplementation by comparing the pre-dialysis concentration of lipid hydroperoxides in plasma and paraoxonase 1 (PON1) activity before and after AA supplementation [58]. PON1 is an HDL-associated enzyme that can preserve low-density lipoprotein (LDL) particles from oxidative changes through the hydrolysis of oxidized lipids [59]. The study results showed that AA supplementation caused a substantial increase in PON1 activity and a decrease in lipid hydroperoxides concentration in plasma from 6.7 ± 0.5 μmol/L to 4.9 ± 0.4. However, the authors did not reveal the utilized dialysis membrane’s type, which can differ in its biocompatibility [6,7].

Nevertheless, AA-related augmentation of the inverse proportionality between the PON1 activity and the levels of lipid hydroperoxides found in these patients manifested the possible relation between PON1, AA, and lipid hydroperoxides as a biochemical index in future dialysis treatment approach [58]. This possible implication would be fulfilled if the authors measured parallel changes of AA levels, which was not done. Placebo control would also strengthen this study.

The issue of lipid peroxidation was further analyzed in a randomized placebo-controlled trial conducted by Abdollahzad et al. The authors studied the effect of oral supplementation of AA compared to placebo (Table 3). The results showed that mean MDA concentration in plasma decreased significantly in the AA treated group from 3.4 ± 1.8 to 2.7 ± 2.0 nmol/mL, while AA level in this group increased. MDA levels maintained steady concentration in the placebo group [60]. AA displayed its protective features against lipid peroxidation, although in this study again, there is no information about the type of dialysis membranes used. 

Observation of lipid peroxidation intensity changes occurring with the initiation of renal replacement therapy is an intriguing matter. The majority of patients with end-stage chronic kidney disease undergo a switch from symptomatic uremia to maintenance hemodialysis. It would be interesting if AA can minimize treatment-related oxidative stress in these patients [2]. Ramos et al. [61] decided to study this relationship in hemodialysis-naive patients with end-stage chronic kidney disease who would begin maintenance hemodialysis (Table 3). The study evaluated the effect of 1 g of AA daily versus placebo administered for one year after hemodialysis initiation on the concentration of thiobarbituric acid reactive substances in LDL plasma fraction. The results showed an increase in TBARS-LDL concentration in both groups, but this was significantly smaller in the AA group compared to the placebo. TBARS-LDL levels (in nanograms per gram LDL) in the placebo and AA supplemented group raised from 0.3 ± 0.2 to 0.5 ± 0.2 and from 0.3 ± 0.2 to 0.4 ± 0.2, respectively. This study’s findings suggest that continuous oral supplementation of relatively large AA doses inhibits the hemodialysis-related oxidation of lipids. However, it has two questionable points; (1) the number of patients in each group (intervention vs. placebo) was not revealed, (2) only median time of AA/placebo administration in the study group was disclosed, which suggests an unequal time of AA treatment among patients [61]. These two limitations can severely affect the results, depending on the extent of mentioned variables, which undermines the study’s credibility. Moreover, AA concentration in plasma was not measured, which could have been done to ascertain AA-dependence of obtained results. 

General effects of AA supplementation on oxidative stress can be measured in plasma, but more specific consequences refer to certain cellular subpopulations, such as erythrocytes. As hemodialysis patients struggle with anemia, any approach which can improve erythrocyte survival is desirable. Ruskovska et al. (Table 3) observed in their study that 1 g of oral AA supplementation taken daily for four weeks increased plasma antioxidant potential measured with ferric reducing ability power (FRAP), from 1182 ± 241 to 1322 ± 306 μmol Fe^2+^/L. Simultaneously, oxidative damage to erythrocyte membranes measured with protein carbonyls concentration in proteins derived from the lysate of erythrocyte membranes was minimized. The concentration of protein carbonyls decreased from 1.0 ± 0.4 to 0.4 ± 0.2 nmol carbonyl/mg protein. Ankyrin was the membrane protein indicated as the most susceptible for oxidative damage, which could be protected by vitamin C [62]. The study’s weaknesses include a low number of patients (*n* = 11), lack of a placebo group, and lack of information about dialysis dose and type of dialysis membranes, which would depict the total time of blood exposure to certain immunogenic artificial materials. An interesting observation of the study was that a single hemodialysis session did not change the concentration of protein carbonyls in erythrocyte membranes while it would have been expected to do so. Therefore, tracking the changes of AA concentration in plasma, introducing the control group, and recording the impact of 4 weeks of dialysis without AA supplementation on protein carbonyl concentration would be valuable. If this has been done, the strength of the study would have increased. 

Inhibition of oxidative damage to erythrocyte membranes’ lipids was also observed by Yang et al. (Table 3), who presented a decrease of phosphatidylcholine hydroperoxide content in erythrocytes after two months of large intravenous doses of AA [57] and by Candan et al. (Table 3), who noticed a decrease in MDA concentration in erythrocytes of 15 patients after 90 days of oral AA supplementation. It was followed by improved membrane strength expressed by reducing hemolysis susceptibility, which took place in 0.42% saline compared to 0.48% saline in the placebo group [10]. Unfortunately, it is difficult to assess if the studied patients were representative enough for hemodialysis patients because the authors did not reveal hemodialysis vintage. Thus, the time of exposure to factors related to this method of treatment remained unknown. 

The other crucial group of cells is circulating lymphocytes because they link inflammatory stimulation and oxidative stress related to hemodialysis. Tarng et al. isolated lymphocytes from peripheral blood before the dialysis session from patients who were receiving AA infusions for the previous eight weeks and observed a considerable reduction of spontaneous and induced generation of ROS compared to placebo (Table 3). ROS reacted with an intracellular probe, leading to light emission, which was recorded [21]. Moreover, as ROS can damage nucleic acids, the concentration of 8-hydroxy-2’-deoxyguanosine (8-OHdG) in lymphocytes’ DNA was measured. 8-OHdG content decreased significantly and inversely correlated with the rise of AA concentration in plasma, underlining its protective influence (Table 3). Dependence of 8-OHdG formation on ROS was shown as the content of the former moderately correlated with the latter (Table 3) [21]. Thus, AA can protect lymphocytes’ DNA either by inhibiting ROS formation or scavenging them. 

Ferric iron infusions are the other factors contributing to oxidative stress in hemodialysis patients; however, they are necessary to treat anemia effectively. Therefore, the information on AA’s capability in minimizing this stress is essential. Conner et al. (Table 3) noticed that if the iron was infused in an interdialytic day together with 300 mg of AA, a drop in mitochondrial membrane potential in peripheral blood mononuclear cells was observed less frequently than if the iron was infused alone [63]. Loss of mitochondrial membrane potential leads to a decrease of ATP synthesis, shortage of energy in a cell, and accumulation of reducers, leading to cell damage [4]. Therefore, AA, by counteracting those effects, potentially may lengthen white cell lifespan in hemodialysis patients. However, the authors did not measure AA concentration in plasma to confirm this relationship. The type of dialysis membranes regarding their biocompatibility was not revealed, which is another weak point of the study. 

Increased oxidative stress hampers the endothelium and intracellular signaling function, which impairs the relaxation of arteries and decreases blood flow. A single oral dose of 2 g of AA increased the average flow-mediated dilation of the brachial artery in 20 hemodialysis patients by 4.7% [14]. This observation links antioxidant features of AA with the effects on arterial endothelium and arterial smooth muscle tension. 

The studies presented above allow us to conclude that oral and intravenous AA supplementation can minimize oxidative stress in hemodialysis patients, highlighting the beneficial effects of vitamin C through its antioxidant and free-radical scavenging properties. AA inhibited the formation of H_2_O_2_ and other forms of ROS in blood and particularly in lymphocytes and protected the latter from excessive DNA damage. AA limited lipids peroxidation, especially in erythrocytes and in LDL plasma fraction; moreover, it stimulated paraoxonase-1 activity. One study reported AA dependent dilation of the brachial artery. Nevertheless, some of those studies have various weaknesses, for instance: a low number of participants or, in one case, unknown method of their randomization, missing information about hemodialysis vintage, dose or dialysis membranes, absence of application of placebo, and lack of measurements of AA concentration. These drawbacks partially question their credibility.

**Table 3:** The inhibitory effect of ascorbic acid supplementation on oxidative stress in hemodialysis patients.

Objective	Study Description	Main Results	References
Assessment of the influence of oral AA supplementation on peroxidative damage of lipids in erythrocytes’ membranes.	Prospective, open label, randomized, placebo-controlled trial, 34 HD patients (mean age 46, HD vintage unknown, dose 12 h/week) received orally 250 mg of AA/day for 90 days (*n* = 15) or placebo. MDA content in erythrocytes and their osmotic fragility was measured in pre-HD samples.	Together with the increase of plasma AA concentration osmotic fragility of erythrocytes decreased by 13%. MDA concentration decreased by 18%. No changes were observed after placebo.	Candan et al. (2002) [10]
Evaluation of the influence of intravenous AA supplementation on ROS production and DNA damage in lymphocytes.	Prospective, open label, randomized, placebo-controlled trial, 51 HD patients (mean: age 59, HD vintage 46 months, cellulose membranes, HD dose 12–13.5 h/week) received 300 mg of AA or saline (*n* = 26) after HD for 8 weeks. ROS production was measured with DCF luminescence, DNA damage with 8-OHdG content in lymphocytes isolated from pre-HD blood samples.	Spontaneous and PMA- stimulated ROS production became 5 and 2 fold smaller respectively with no changes after placebo. 8-OHdG content decreased by 18% and inversely correlated with the increase in AA concentration (r= −0.65), while directly with spontaneous and PMA-stimulated ROS production (r= 0.49 and 0.63 respectively).	Tarng et al. (2004) [21]
Studying the short and long term effects of IV infusion of AA during HD sessions on HD-related oxidative stress.	Prospective, observational, open label study, 20 HD patients in the intervention and 20 in the control group, Kt/V: 1.2–1.5, Polysynthane membranes, mean HD vintage 12 months. 1000 mg of AA was infused during each HD-session for 2 months.	AA suppressed HD-related ROS formation in blood by 86%, compared to no-intervention, inhibited the rise in H_2_O_2_ content in plasma by 60% and phosphatidylcholine hydroperoxide in plasma and erythrocytes by 45% and 35% respectively.	Yang et al. (2006) [57]
Studying the effect of oral AA supplementation on LDL oxidation.	Randomized, placebo-controlled, open label trial, 34 patients initiating HD (mean age 57, cellulose acetate membranes) received 1 g/d of AA or placebo for median of one year, the number of patients in each group is unknown.	Oxidized TBARS-LDL concentration increased more in the placebo group (by 64% of initial value) than in the AA group (52%).	Ramos et al. (2008) [61]
Studying the effect of intravenous AA supplementation on lipid peroxidation and PON1 activity.	Prospective Observational Study, 33 HD patients (mean: age 66, HD vintage 80 months, HD dose 12 h/week, membrane type unknown) received 500 mg of AA IV thrice a week for 6 months. PON1 activity and lipid hydroperoxides concentration in pre-HD plasma was measured.	PON1 activity increased by 60% of initial value, lipid hydroperoxides concentration decreased by 25%.	Ferretti et al. (2008) [58]
Studying the effects of oral AA supplementation on lipid peroxidation.	Randomized, placebo-controlled, double blinded trial, 42 HD patients (mean age 60, HD vintage 6 years, HD dose 12 h/week, membranes type unknown) received 250 mg of AA thrice a week for 12 weeks (n =21) or placebo. Measurements were performed in Pre-HD plasma.	MDA concentration decreased by 20% of initial value, while AA increased by 36%. MDA concentration did not change after placebo.	Abdollahzad et al. (2009) [60]
Assessment of the modification of ferric infusion-dependent oxidative stress by infusion of AA.	Prospective, randomized, open-label, crossover study, 13 HD patients (mean age: 58, HD vintage 74 months, Kt/V 1.6, ferritin 703 ng/mL, HD membranes unknown). 100 mg of ferric sucrose alone (IS group) or with 300 mg of AA (IS + C group) was infused in interdialytic day separated by 2 week wash-out period. PBMC were subsequently isolated.	PBMC from 13 patients in the IS group compared to 7 patients in the IS + C presented loss of mitochondrial membrane potential.	Conner et al. (2012) [63]
Studying the effect of oral AA supplementation on erythrocytes’ membrane proteins oxidation and plasma FRAP.	Prospective observational study, 11 HD patients (mean HD vintage 6 years, HD dose and membranes type unknown), 1000 mg of AA a day was administered enterally for 4 weeks. Pre-HD blood samples were collected.	Plasma FRAP increased by 12%. Total erythrocytes’ membrane protein carbonyls decreased by 63% of the initial value.	Ruskovska et al. (2015) [62]

8-OHdG 8-hydroxy-2’-deoxyguanosine; AA, Ascorbic Acid; DCF, dichlorofluorescein; FRAP, Ferric reducing ability power; HD, Hemodialysis; IV, Intravenous; Kt/V, parameter of dialysis dose; LDL, low-density lipoprotein; MDA, malondialdehyde; PBMC, peripheral blood mononuclear cells; PMA, phorbol myristate acetate; PON1, paraoxonase 1; ROS, reactive oxygen species; TBARS, thiobarbituric acid reactive substances.

## 7. Inhibitory Effects of Ascorbic Acid Supplementation on the Intensity of Inflammation among Hemodialysis Patients 

One of the causes of oxidative stress and cardiovascular diseases in hemodialysis patients is chronic inflammation characterized by the elevation of C-reactive protein (CRP) levels [64,65]. A randomized placebo-controlled trial conducted by Biniaz et al. (Table 4) presented a decrease in CRP concentration in a group of 141 hemodialysis patients after eight weeks of AA administration. 250 mg of AA thrice-weekly reduced median CRP concentration in serum from 16.8 ± 27.9 to 10.8 ± 25.4 mg/L, whereas it increased in both the placebo and no intervention groups from 17.8 ± 27.6 to 22.6 ± 38.5 mg/L and from 19.4 ± 26.7 to 30.7 ± 46.4 mg/L, respectively. These findings show anti-inflammatory features of vitamin C, although correlating changes of its concentration were not measured. A severe limitation of the study, which undermines the significance of obtained results, is the absence of information about the specific time frame of blood sample collection [66]. The uncertainty of whether the CRP levels were determined pre- or post-hemodialysis session has important implications as dialysis sessions can independently increase CRP concentration [64,67]. Moreover, information about the type of dialysis membranes used in the study is missing; different membrane materials present various biocompatibility levels and the ability to stimulate inflammation and CRP synthesis [67]. 

More precisely, Baradari et al. (Table 4) addressed the same purpose of the previous study, but on a smaller group of 58 patients dialyzed with polysulphone membranes. The authors found that 500 mg of AA administered intravenously decreased CRP concentration in pre-HD serum from 11 ± 8.2 mg/dL to 7.3 ± 6.8 mg/dL while no changes were observed in placebo group. This study also indicates AA as an agent, slightly but significantly, inhibiting inflammation in hemodialysis patients [68]. However, changes in AA concentration were not measured to confirm the observed relationship. Study results would not provoke any doubts if the measured CRP concentration were expressed in the correct units, which should be mg/L instead of mg/dL unless the patients had a severe inflammatory disease, but this was instead not the case. 

Zhang et al. noted that AA level in plasma of hemodialysis patients presented a negative correlation with the concentration of high sensitivity C-reactive protein (hs-CRP) (r = −0.20) and positive with pre-albumin and albumin concentration (r = 0.27 and 0.16 respectively) [46]. Albumin and pre-albumin are negative markers of inflammation. A more recent study searched for a target where observed abnormalities could be intensely dependent on AA concentration (Table 4). The authors selected hemodialysis patients with a low AA concentration (median 10 μmol/L) and concomitant chronic inflammation characterized by hs-CRP concentration elevated above 3 mg/L [54]. The increase of this inflammatory marker above that level is associated with a higher risk of cardiovascular diseases, especially in patients with additional risk factors such as end-stage chronic kidney disease [69]. In this randomized cross-over trial on 100 patients divided into two groups, Zhang et al. assessed the impact of a 200 mg oral dose of AA per day on the hs-CRP pre-dialysis concentration. This study showed that median hs-CRP concentration dropped from 9.6 to 4.9 mg/L in the first group and 6.2 to 5.1 mg/L in the second and returned to baseline after withdrawal. Concomitantly, the measured rise in plasma concentration of AA confirmed the inverse relationship between this compound and the concentration of hs-CRP [54], displaying anti-inflammatory and consequent cardiovascular-protective properties of vitamin C. The study has two weak points; although it was performed in a cross-over manner, a placebo was not applied, and information about the type of dialysis membrane was missing. 

In conclusion, the results of mentioned studies revealed the anti-inflammatory characteristic of vitamin C presented by the decrease of CRP levels in hemodialysis patients after AA supplementation. Following this approach to managing end-stage chronic kidney disease, hemodialysis patients may be protected against inflammation-induced cardiovascular events. However, several studies suggest that this anti-inflammatory effect is small, and some of them have the following drawbacks: lack of monitoring of AA concentration in patients’ blood, missing information about the type of dialysis membranes, one study was not controlled by the placebo, one did not reveal time relationship between dialysis session and blood collection, the other expressed CRP concentration in faulty units. These weaknesses decrease the strength of the presented studies.

## 8. Unfavorable Effects of Ascorbic Acid Supplementation on Oxidative Stress and Inflammation in Hemodialysis Patients

Studies presented above revealed that ascorbic acid supplementation in hemodialysis patients precluded lipid peroxidation. On the contrary, Chen et al. (Table 5), in a study done on 29 hemodialysis patients, found ascorbic acid acting as a pro-oxidant after a parenteral administration of a single bolus of 300 mg, compared to a placebo. Free radical generation was determined by lucigenin-enhanced chemiluminescence (LucCL) assay on blood samples collected during dialysis sessions and ascorbate or placebo administration. After a bolus of ascorbic acid, LucCL intensity was significantly higher than in patients who received placebo (1261.0 ± 401.9 vs. 77.4 ± 62.5 relative light unit, RLU) [20]. Surprising pro-oxidant features of an otherwise known antioxidant can be explained by the fact that vitamin C catalyzes ferric iron’s transformation to ferrous iron [70]. Ferrous iron can act as a pro-oxidant by enhancing hydroxyl radical formation in Fenton’s reaction, a significant ROS player [71]. In Chen’s study, patients had a high mean concentration of ferritin in plasma (856.9 ng/mL), a protein containing ferric iron.

In hemodialysis patients, the route of choice for ferric iron administration is intravenous due to its low absorption in intestines and the need to preserve proper serum iron levels. There is a possibility that iron infusions aggravate oxidative stress if administered simultaneously with intravenous AA. This issue was investigated by Eiselt et al., who studied the rise of thiobarbituric acid reactive substances in plasma after ferric sucrose infusion during hemodialysis in 20 patients (Table 5) who initially were AA deficient. After a month of oral AA supplementation and reaching the normal levels of AA in plasma, TBARS measured in plasma after repeated hemodialysis sessions with parallel ferric sucrose infusion remained at the same level as before the supplementation [38]. When ferric sucrose infusion was combined with simultaneous AA infusion, TBARS concentration in plasma increased a little more (from 2.1 to 3.1 μmol/L) than in the case of ferric infusion alone (from 1.5 to 2.6 μmol/L). These results can be interpreted as evidence of AA’s weak pro-oxidant action in the iron-containing environment [38]. 

In combination with ferrous iron and other metals, AA acts as a potential oxidant in various chemical and enzymatic systems in vitro [27,28]. Reactions of AA with ferric ion could be influential as Chen et al. observed a strong significant correlation between the intensity of blood LucCL and ferritin concentration in serum (Table 5) [20]. In patients with a ferritin level higher than 600 ng/mL, ROS generation was 5.5-fold more intense than in patients with its concentration below this level. However, the hemodialysis vintage of studied patients was not revealed, making it difficult to assess if participants were representative enough for hemodialysis patients. In the in vitro part of the research, the authors noticed a significant inhibition of ROS synthesis by the iron chelator deferoxamine, which confirms iron’s crucial role [20]. 

Conner et al. (Table 5) compared the effects of simultaneous infusion of AA and ferric iron with ferric iron alone on an interdialytic day in 13 hemodialysis patients. Simultaneous infusion of both agents resulted in higher levels of F2-isoprostanes compared to infusion of iron alone [63]. F2-isoprostane is a marker of oxidative stress related to inflammation and lipid peroxidation [72]. In parallel, the authors noticed an increase in concentrations of inflammatory markers in plasma, such as interleukins 1 and 10 and tumor necrosis factor-α, which were not observed after infusion of iron alone. Moreover, simultaneous AA and iron administration stimulated peripheral blood mononuclear cells to generate higher amounts of superoxide anion [63]. These observations indicate AA as a pro-oxidant and pro-inflammatory agent if administered intravenously together with ferric iron. 

Washio et al. (Table 5) demonstrated that oral supplementation of vitamin C in an increasing dose led to a growth in its plasma concentration and its oxidized form [19]. The increase of the oxidized form of ascorbic acid in plasma was related to the dialysis session. Once ascorbic acid was oxidized, other substances like ferric ions could be reduced. The resultant ferrous ion could initiate subsequent pro-oxidant reactions. Moreover, AA’s enzymatic regeneration from dehydroascorbic acid (oxidized form) involves oxidation of glutathione and decreases GSH/GSSG ratio [18]. The Washio et al. study also showed that vitamin C did not affect copper/zinc superoxide dismutase (Cu/Zn-SOD) concentration in plasma and expression in leukocytes [19]. The enzyme utilizing toxic superoxide anion was expected to present decreased activity if ascorbic acid scavenged the anion in concurrent reactions, but it did not take place. The dose of hemodialysis applied in this study and type of dialysis membranes remain unknown, hiding the strength of pro-inflammatory factor related to the length of contact with a specific membrane. The study was not controlled with placebo.

Chronic intravenous administration of 100 mg of vitamin C after each hemodialysis session did not prevent the formation of advanced oxidative protein products and advanced glycation end products (AGEs) in hemodialysis patients dialyzed with polysulphone membranes. Patients presented 2.3- and 2.8-fold higher concentrations of AOPP and AGE, respectively, than healthy subjects, after adjusting for albumin and triglyceride concentrations, in De Mattos et al.’s cross-sectional study [73]. On the other hand, one could tell that the observed markers’ concentration could be even higher if AA would not have been infused, which can be a true statement in AOPP only. This research showed a direct moderate correlation between AGEs and ascorbic acid concentrations in serum (r = 0.46), indicating the latter as a possible perpetrator of AGEs formation [73]. Certain studies demonstrated that under oxidative stress, oxidized ascorbic acid which concentration increases during hemodialysis sessions [19], might be a precursor of AGEs via 2,3-diketogulonic acid [74]. AGEs, in turn, can bind to their receptor (RAGE) in various cells leading to increased inflammatory response and consequent oxidative stress [75]. De Mattos’s study limitations include its cross-sectional character only and lack of intervention; for instance, the cross-over manner would more reliably prove AA’s influence on measured parameters. Instead, studied patients had been supplemented with AA continuously before and during the study. The other weakness is that it relies on the spectrophotometric method of AA determination, which is less precise than HPLC. 

Considering LDL oxidation, when vitamin C was added to the reaction mixture in vitro after the initiation of LDL oxidation with CuCl2, it enhanced this process [76]. It corresponds with in vivo study by De Vriese et al. on 92 hemodialysis patients (Table 5) who received increasing oral AA doses for six months. The authors observed an analogous dose-dependent rise of plasma malondialdehyde concentration. The increase reached 26% and positively correlated with the rise of AA concentration in plasma (r = 0.4) [53]. This study points out the most considerable oxidative stress level measured with MDA found in hemodialysis patients with the highest plasma levels of AA. MDA decreased three months after vitamin C was withdrawn [53]. Independently, MDA concentration and the rate of its relative increase during vitamin C therapy, but not the MDA baseline level, correlated moderately with serum ferritin levels (r = 0.4) [53], which again marks the critical role of iron in vitamin C-related oxidative stress. The ferritin concentration above 500 μg/L tripled the risk of elevation of MDA concentration in serum above 2 μmol/L while the baseline concentration was 1.5 μmol/L [53]. Up to 40% of HD patients can have serum ferritin elevated above that level; therefore, this observation has profound implications [77]. In the authors’ opinion, the possible benefits of supplementing patients on hemodialysis with ascorbic acid are domineered by the adverse effects of lipid peroxidation promoted this way [53].

A double-blind placebo-controlled trial conducted by Kamgar et al. (Table 5) on 37 hemodialysis patients showed that a cocktail of high-dose oral antioxidants administered every day for eight weeks failed to correct the level of oxidative stress in patients maintained on hemodialysis. The high-dose oral antioxidant mixture enclosed vitamin E, vitamin C (250 mg), B6, B12, and folic acid. Neither the products of lipid and protein peroxidation in plasma, f-2 isoprostane, and protein carbonyls, nor the levels of inflammatory markers: CRP and interleukin-6, were affected by the intervention. The dose of hemodialysis applied in the study remains unknown [77]. The authors did not track AA concentration in plasma, which would have confirmed patients’ compliance. 

No influence on serum albumin concentration, a negative marker of inflammation, was observed in 100 patients treated orally with 200 mg of AA a day despite the elevation of AA level in plasma [54]. Similarly, no effect on hs-CRP and albumin in plasma was observed after oral supplementation of 250 mg of AA every second day for two months, although AA’s concentration increased [17]. The same group of 33 patients did not present any change in markers of oxidative stress, chosen by Fumeron et al. (Table 5), which were concentration of protein carbonyls, oxidized to reduced glutathione (GSSG/GSH) ratio in erythrocytes, and oxidized to reduced vitamin C ratio in plasma [17]. However, the study was not controlled by the placebo. Similarly, Chan et al. (Table 5) observed no effect of enteral or intravenous AA supplementation in a dose of 250 mg after each hemodialysis session on F2-isoprostanes concentration in plasma of 21 hemodialysis patients, although they had slightly elevated ferritin concentration [78]. The authors of this study did not provide information about the membrane used, which can influence the extent of hemodialysis-related inflammation. 

Eventually, in contrast to the relevant study presented in the previous chapter [14], Cross et al. found no effect of a single intravenous bolus of 3 g of vitamin C on flow-mediated dilation of the brachial artery in 17 hemodialysis patients, denying the link between the inhibition of oxidative stress and the improvement of arterial blood flow which otherwise could be attributed to AA [79]. Current data about AA remains controversial regarding hemodialysis patients and the general population [80,81].

To conclude, the mentioned studies’ results showed a lack of effects or an opposite effect of AA supplementation in hemodialysis patients compared to the previous chapters. The pro-oxidant aspect of AA supplementation was presented by increasing the free radical generation, lipid and protein peroxidation markers. The pro-inflammatory effect was marked by increased concentrations of specific cytokines and advanced glycation end-products related to AA. Higher levels of ferritin in plasma precipitate unwanted effects of AA. Co-administration of AA with ferric iron remarkably aggravated oxidative stress and inflammation, and this aspect is undeniable. Certain studies have weaknesses, as they were not controlled with placebo—one was cross-sectional, others did not provide information about hemodialysis vintage, dose or dialysis membrane or included few patients. 

**Table 5:** Studies demonstrating no effect or unfavorable effects of vitamin C supplementation on oxidative stress or inflammation in hemodialysis patients.

Objective	Study Description	Main Results	References
Assessment of the oxidative stress changes related to intravenous AA infusion.	Placebo-controlled, open label, randomized trial, 29 HD patients (mean: age 64, HD vintage unknown, Kt/V 1.4, Excebrane membranes). 300 mg of IV AA (n = 18) or saline was administered at the beginning of HD session. Blood samples were collected shortly before and after the injection for LucCL assay.	LucCl in the AA group was 16-fold higher than in placebo group, and correlated with ferritin concentration (r^2^ = 0.87).	Chen et al. (2003) [20]
Evaluation of the effect of oral AA supplementation on plasma and erythrocyte markers of oxidative stress.	Prospective, randomized, observational study, 33 HD patients (mean: age 52, HD vintage 6.1 years, Kt/V 1.2, cellulose diacetate and polysulfone membranes) received 250 mg of AA orally after each HD for 2 months or no drug. Plasma concentrations of DHA/AA, albumin, hs-CRP, protein carbonyls, erythrocyte GSH/GSSG.	Despite the elevation of AA concentration levels of measured markers remained unchanged.	Fumeron et al. (2005) [17]
Studying the influence of oral or IV AA supplementation on concentration of F_2_-isoprostanes in plasma of HD patients with hyperferritinemia	Prospective, randomized, parallel observational study, 21 HD patients (mean: age 56, HD vintage > 6 months, serum ferritin 632 μg/L, sessions thrice a week, URR 76%, HD membrane unknown) received 250 mg of AA orally or intravenously after HD sessions for 8 weeks. F_2_-isoprostanes were measured in pre-HD plasma.	Despite the elevation of AA concentration in plasma, no changes of F_2_-isoprostanes concentration were observed.	Chan et al. (2006) [78]
Evaluation of the modification of HD and ferric iron infusion-dependent oxidative stress by oral and intravenous AA.	Placebo-controlled, open-label, randomized trial, 20 AA deficient HD patients (mean age: 73, HD vintage 39 months, Kt/V 1.4, polysulphone membranes). 100 mg ferric sucrose was infused in AA deficient patients during HD, then 250 mg of AA a day for 2 weeks and 50 mg/day for the next 2 weeks was supplemented orally, and ferric sucrose or saline or AA or both were infused during HD and blood samples were taken. AA was measured with HPLC.	Ferric sucrose infusion induced equal increase in plasma TBARS in AA deficient or non-deficient patients. TBARS raised by 44% when iron was infused alone or by 47% when combined with AA.	Eiselt et al. (2006) [38]
Evaluation of the effects of oral administration of AA-containing antioxidant cocktail on plasma/serum concentration of markers and mediators of oxidative stress and inflammation.	Cross-sectional at baseline, longitudinal with 8-week follow-up, double-blinded, placebo-controlled trial, 37 HD patients (mean: age 52, HD vintage 54 months, HD dose unknown, cellulose acetate membranes); 20 treated every day with cocktail containing 250 mg of AA. Concentrations of f-2 isoprostane, protein carbonyls, CRP and IL-6 in pre-HD plasma was measured.	Concentration of f-2 isoprostane, protein carbonyls, CRP, and IL-6 remained unaffected after the treatment.	Kamgar et al. (2007) [77]
Studying the effect of oral AA supplementation on MDA concentration in plasma.	Prospective observational study. 92 HD patients (mean: age 67, HD vintage 2.9 years, Kt/V 1.4, cellulose diacetate membranes). AA was administered orally, after each HD session first at a dose of 360 then 1500 mg/week for 3 months. MDA was quantified in pre-HD plasma.	Mean MDA concentration increased from 1.5 ± 0.4 μmol/L to 1.6 ± 0.5 and 1.9 ± 0.5 μmol/L after 3 and 6 months respectively. Parallel rise of AA concentration is presented in Table 2.	De Vriese et al. (2007) [53]
Assessment of the effects of orally administered AA on plasma activity and concentration of Cu/Zn-SOD and its expression in leukocytes.	Prospective observational study 16 HD patients (mean: age 64, HD vintage 7.9 years, HD dose and membranes unknown). 1st month of the study: oral administration of 200 mg of AA 1 h before HD sessions. 400 mg and 1000 mg of AA were given during the 2nd and the 3rd month, respectively. The levels of Cu/Zn-SOD in pre-HD plasma and its mRNA expression in leukocytes were determined.	No changes in plasma Cu/Zn-SOD concentration and activity or its mRNA expression were observed. The parallel rise of AA concentration is presented in Table 2.	Washio et al. (2008) [19]
Assessment of the modification of iron infusion-dependent oxidative stress and inflammatory response by infusion of AA.	Prospective, randomized, open-label, crossover study, 13 HD patients (mean age: 58, HD vintage 74 months, Kt/V 1.6, ferritin 703 ng/mL). 100 mg ferric sucrose alone (IS group) or with 300 mg of AA (IS + C group) was infused in interdialytic day separated by 2 week wash-out period. Blood samples for IL-1, IL-10, TNF-α, F_2_-isoprostanes and PBMC for the assessment of intracellular O_2_^−^. and H_2_O_2_ generation were taken.	Concentrations of: IL-1, IL- 10, TNF-α, F_2_-isoprostanes rose 2.4, 1.4, 1.8, 1.2 fold respectively in the IS + C group but not in the IS group. O_2_^−^. generation increased 2.4 fold more in the IS + C group than in the IS. H_2_O_2_ generation did not differ between groups.	Conner et al. (2012) [63]
Studying the effect of oral AA supplementation on serum albumin concentration in HD patients with low initial AA level and high hs-CRP level.	Prospective, randomized, cross-over, observational study, 100 HD patients (mean: age 64, HD vintage 48 months, Kt/V 1.5, membrane type unknown), AA concentration in plasma < 4 μg/mL *. Equal groups received 200 mg/day of AA orally or nothing for 3 months. Measurements were made in pre-HD plasma.	Albumin concentration was not influenced by AA supplementation despite the rise of AA plasma level (Table 2).	Zhang et al. (2013) [54]
Studying the relation between serum AA concentration and markers of oxidative stress (AOPP, AGEs) and inflammation (CRP, IL-6) in HD patients on intravenous AA supplementation.	Cross-sectional study, 21 HD patients (mean: age 54, HD vintage 45 months, Kt/V 1.7, polysulfone membranes) received 100 mg of AA thrice a week. Concentrations of markers were measured in pre-HD serum, AA with spectrophotometry.	AGEs concentration showed a correlation with AA concentration (r = 0.46). Other parameters did not correlate with AA.	Marques de Mattos et al. (2014) [73]

* Conversion factor for units: vitamin C μg/mL to μmol/L × 5.678. AA, ascorbic acid; AGEs, advanced glycation end-products; AOPP, advanced oxidation protein products; CRP, C-Reactive Protein; DHA, dehydroascorbate (oxidized); Cu/Zn-SOD, copper/zinc superoxide dismutase; GSH, glutathione; GSSG, glutathione disulfide (oxidized); hs-CRP, high-sensitivity C-reactive protein; HD, hemodialysis; IL-1 interleukin 1, IL-6, interleukin 6; IL-10 interleukin 10, IV intravenous; Kt/V, parameter of dialysis dose; LucCL, lucigenin-enhanced chemiluminescence; MDA, malondialdehyde; PBMC, peripheral blood mononuclear cells; TBARS, thiobarbituric acid reactive substances; TNF-α, tumor necrosis factor α; URR, urea reduction ratio.

## 9. Safety of Ascorbic Acid in Hemodialysis Patients

Although most of the presented studies did not report any clinically symptomatic adverse effects of vitamin C supplementation, its above-mentioned specific prooxidant effects cannot be neglected. Also, very few studies monitored the concentration of oxalate in plasma, a potentially toxic metabolite of AA. Yang et al. measured the concentration of oxalate in hemodialysis patients’ plasma and found no changes of this parameter after infusions of 3000 mg of AA a week. However, those measurements were performed shortly after infusion; therefore, AA did not have time to undergo degradation to oxalate [57]. Another study reported an insignificant increase in pre-dialysis plasma oxalate after intravenous administration of 300 mg of AA after each dialysis [50]. On the other hand, Canavese et al. observed a significant increase in plasma oxalate concentration, which depended on a dose of infused AA. However, changes in AA concentration in plasma presented only weak correlations with changes of oxalate concentration (r = 0,2). After 12 months of intravenous supplementation of AA (to AA deficient patients), at a dose of 500 mg a week, AA concentration more than tripled. Meanwhile, oxalate concentration crossed the supersaturation level recognized as 50 μmol/L in 6 of 16 patients (38%) than 1 of 18 at baseline (5.6%).

A similar phenomenon of increased oxalate concentration was observed in other studies among adults and children on hemodialysis after prolonged enteral or intravenous AA supplementation [51,55]. The fact of exceeding saturation level brings a risk of oxalate deposition in tissues, mainly vessels, and bones. Paradoxically, such a result of vitamin C overdosage would possibly precipitate the occurrence of cardiovascular diseases instead of preventing them [37]. The risk of crossing this frontier can be smaller if AA is administered orally because of various regulatory mechanisms of vitamin C absorption and elimination. In case of an increase in the dose, oral administration limits AA bioavailability and ensures a certain kind of pharmacokinetics saturation [22,25]. After a gradual increase in the enteral dose over 200 mg/day up to 2500 mg/day, the mean concentration of AA in plasma of 22 healthy persons did not exceed 80 μmol/L and remained steady through the doses [22]. The maximum individual concentration observed after the enteral administration of 1250 mg of vitamin C rose relatively slowly and never exceeded 200 μmol/L.

On the other hand, intravenous administration of the same dose in healthy subjects resulted in concentrations exceeding 1000 μmol/L [22]. The sudden peak of ascorbic acid, followed by its non-enzymatic breakdown, can lead to an intense oxalate generation [22]. Thus, hemodialysis patients on intravenous AA supplementation displayed a tendency to higher AA and oxalate levels in plasma than the ones supplemented orally [55]. Nevertheless, oxalate-related, biopsy-confirmed, two cases of bone destruction manifesting with fractures or deformations were recently reported in hemodialysis children after long-term uncontrolled enteral supplementation of vitamin C [51]. Moreover, in adults, a longer dialysis time was associated with a more frequent finding of calcium oxalate deposition in biopsies of subsequently transplanted kidneys, accompanied by a delayed graft function [82,83]. This situation may result in a consequent decrease in glomerular filtration in transplanted kidney or patient’s death [83]. One cannot exclude the link between post-transplantation oxalosis and AA overdosage before transplantation, during life on maintenance hemodialysis.

Concluding, uncontrolled or excessive supplementation of AA in hemodialysis patients may result in symptomatic oxalosis.

## 10. Conclusions

The majority of studies show an increased frequency of AA deficiency in hemodialysis patients compared to healthy subjects, two studies associated this condition with the increase in patients’ mortality. Many experiments present beneficial effects of oral or intravenous supplementation of AA, concerning the effectiveness in counteracting the deficiency and exhibiting an approach against oxidative stress, chronic inflammation, and cardiovascular diseases. However, these studies’ results are not strong enough to disprove the results of the experiments that present prooxidant and proinflammatory actions of AA. All beneficial effects of AA supplementation were undermined by the studies showing opposite actions with certain following exceptions. One research showed the protective effects of AA against DNA mutations in lymphocytes, and another revealed stimulation of paraoxonase-1 activity in plasma. On the other hand, the increase in proinflammatory advanced glycation end-products in serum related to AA administration was also not denied by any other study. Investigations of AA’s impact on arterial flow presented either its improvement or lack of any effect. Deleterious effects of AA supplementation were significantly associated with the presence of iron, either endogenous bound to ferritin or exogenous. None of the studies presented any beneficial effect of AA in the environment containing relatively high iron concentrations. 

Both “pro-AA” and “anti-AA” studies have specific weaknesses, but their extent is not enough to accept one study’s superiority over the others. None research exceeded the number of 150 participants, and most were much smaller. One recent study reported clinically apparent, serious, unwanted effects of vitamin C supplementation in children on hemodialysis related to oxalosis. The question of; to recommend or not recommend Vitamin C supplementation for dialysis patients remains continuously debatable. AA administration in hemodialysis patients may be beneficial if its initial plasma concentration is lower than 30 μmol/L. A physician who makes a decision, obeying the safety-first rule, should take into consideration: concentration of AA in plasma and its relation to the reference values, the concentration of ferritin in serum, which is better to be lower than 500 μg/L, avoidance of simultaneous iron infusion, route of AA administration with the preference of enteral one, and avoidance of repeated large doses.

## Figures and Tables

**Table 4:** The inhibitory effect of ascorbic acid supplementation on the intensity of inflammation in hemodialysis patients.

Objective	Study Description	Main Results	References
Studying the effect of intravenous AA supplementation on serum CRP concentration.	Randomized, double blinded, placebo-controlled trial, 58 HD patients (mean: age 60, HD vintage 30 months, 12 h of HD a week, polysulphone membranes) received 500 mg of AA IV after HD sessions, for 8 weeks (*n* = 29) or saline. Pre-HD serum was analyzed.	CRP concentration decreased by 34% remaining unchanged after placebo.	Baradari et al. (2012) [68]
Studying the effect of oral AA supplementation on plasma hs-CRP level in AA deficient patients.	Prospective, randomized, cross-over, observational study, 100 HD patients (mean: age 64, HD vintage 48 months, Kt/V 1.5, membrane type unknown), initial AA concentration in plasma < 4 μg/mL *. Equal groups received 200 mg/day of AA orally or no drug for 3 months. Pre-HD plasma was analyzed.	Hs-CRP concentration decreased by 28 to 49%. AA concentration in plasma raised (Table 2).	Zhang et al. (2013) [54]
Studying the effect of intravenous AA supplementation on CRP concentration in serum.	Randomized, placebo-controlled, double blinded trial, 141 HD patients (mean: age 61, HD vintage 40 months, HD sessions 3 × 3.8 h, membrane type unknown) Equal groups received 250 mg of AA IV after HD for 8 weeks or saline or nothing. It is unknown if serum CRP was measured pre- or post-HD session.	Median CRP concentration decreased by 36% in the AA group while it increased by 27% and 58% in the placebo and control group respectively.	Biniaz et al. (2014) [66]

* Conversion factor for units: vitamin C μg/mL to μmol/L × 5.678. AA, Ascorbic Acid; CRP, C-reactive protein; HD, Hemodialysis; hs-CRP, high-sensitivity C-reactive protein; IV, Intravenous; Kt/V, parameter of dialysis dose.

## References

[1] Heaf J. (2017). Current trends in European renal epidemiology. Clin. Kidney J..

[2] Liakopoulos V., Roumeliotis S., Gorny X., Dounousi E., Mertens P.R. (2017). Oxidative Stress in Hemodialysis Patients: A Review of the Literature. Oxid. Med. Cell. Longev..

[3] Granata S., Zaza G., Simone S., Villani G., Latorre D., Pontrelli P., Carella M., Schena F., Grandaliano G., Pertosa G. (2009). Mitochondrial dysregulation and oxidative stress in patients with chronic kidney disease. BMC Genom..

[4] Zorova L.D., Popkov V.A., Plotnikov E.Y., Silachev D.N., Pevzner I.B., Jankauskas S.S., Babenko V.A., Zorov S.D., Balakireva A.V., Juhaszova M. (2018). Mitochondrial membrane potential. Anal. Biochem..

[5] Coaccioli S., Standoli M.L., Biondi R., Panaccione A., Landucci P., Del Giorno R., Paladini A., Standoli M., Puxeddu A. (2010). Open comparison study of oxidative stress markers between patients with chronic renal failure in conservative therapy and patients in haemodialysis. Clin. Ter..

[6] Varan H., Dursun B., Dursun E., Ozben T., Suleymanlar G. (2010). Acute effects of hemodialysis on oxidative stress parameters in chronic uremic patients: Comparison of two dialysis membranes. Int. J. Nephrol. Renovasc. Dis..

[7] Hörl W.H., Steinhauer H.B., Schollmeyer P. (1985). Plasma levels of granulocyte elastase during hemodialysis: Effects of different dialyzer membranes. Kidney Int..

[8] Liakopoulos V., Roumeliotis S., Zarogiannis S., Eleftheriadis T., Mertens P.R. (2019). Oxidative stress in hemodialysis: Causative mechanisms, clinical implications, and possible therapeutic interventions. Semin. Dial..

[9] Heidari B. (2013). C-reactive protein and other markers of inflammation in hemodialysis patients. Casp. J. Intern. Med..

[10] Candan F., Gültekin F., Candan F. (2002). Effect of vitamin C and zinc on osmotic fragility and lipid peroxidation in zinc-deficient haemodialysis patients. Cell Biochem. Funct..

[11] Li D., Mehta J. (2005). Oxidized LDL, a critical factor in atherogenesis. Cardiovasc. Res..

[12] Witko-Sarsat V., Friedlander M., Capeillère-Blandin C., Nguyen-Khoa T., Nguyen A.T., Zingraff J., Jungers P., Descamps-Latscha B. (1996). Advanced oxidation protein products as a novel marker of oxidative stress in uremia. Kidney Int..

[13] Delaney S., Jarem D.A., Volle C.B., Yennie C.J. (2012). Chemical and biological consequences of oxidatively damaged guanine in DNA. Free Radic. Res..

[14] Ghiadoni L., Cupisti A., Huang Y., Mattei P., Cardinal H., Favilla S., Rindi P., Barsotti G., Taddei S., Salvetti A. (2004). Endothelial dysfunction and oxidative stress in chronic renal failure. J. Nephrol..

[15] Deicher R., Ziai F., Bieglmayer C., Schillinger M., Hörl W.H. (2005). Low Total Vitamin C Plasma Level Is a Risk Factor for Cardiovascular Morbidity and Mortality in Hemodialysis Patients. J. Am. Soc. Nephrol..

[16] Ceballos-Picot I., Witko-Sarsat V., Merad-Boudia M., Nguyen A.T., Thévenin M., Jaudon M.C., Zingraff J., Verger C., Jingers P., Descamps-Latscha B. (1996). Glutathione antioxidant system as a marker of oxidative stress in chronic renal failure. Free Radic. Biol. Med..

[17] Fumeron C., Nguyen-Khoa T., Saltiel C., Kebede M., Buisson C., Drüeke T.B., Lacour B., Massy Z.A. (2005). Effects of oral vitamin C supplementation on oxidative stress and inflammation status in haemodialysis patients. Nephrol. Dial. Transplant..

[18] Meister A. (1994). Glutathione-ascorbic acid antioxidant system in animals. J. Biol. Chem..

[19] Washio K., Inagaki M., Tsuji M., Morio Y., Akiyama S., Gotoh H., Gotoh T., Gotoh Y., Oguchi K. (2008). Oral vitamin C supplementation in hemodialysis patients and its effect on the plasma level of oxidized ascorbic acid and Cu/Zn superoxide dismutase, an oxidative stress marker. Nephron Clin. Pract..

[20] Chen W.-T., Lin Y.-F., Yu F.-C., Kao W.-Y., Huang W.-H., Yan H.-C. (2003). Effect of ascorbic acid administration in hemodialysis patients on in vitro oxidative stress parameters: Influence of serum ferritin levels. Am. J. Kidney Dis..

[21] Tarng D.-C., Liu T.-Y., Huang T.-P. (2004). Protective effect of vitamin C on 8-hydroxy-2′-deoxyguanosine level in peripheral blood lymphocytes of chronic hemodialysis patients. Kidney Int..

[22] Levine M., Padayatty S.J., Espey M.G. (2011). Vitamin C: A Concentration-Function Approach Yields Pharmacology and Therapeutic Discoveries. Adv. Nutr..

[23] Levine M., Wang Y., Padayatty S.J., Morrow J. (2001). A new recommended dietary allowance of vitamin C for healthy young women. Proc. Natl. Acad. Sci. USA.

[24] Bendich A., Machlin L.J., Scandurra O., Burton G.W., Wayner D.D.M. (1986). The antioxidant role of vitamin C. Adv. Free Radic. Biol. Med..

[25] Berretta M., Quagliariello V., Maurea N., Di Francia R., Sharifi S., Facchini G., Rinaldi L., Piezzo M., Manuela C., Nunnari G. (2020). Multiple Effects of Ascorbic Acid against Chronic Diseases: Updated Evidence from Preclinical and Clinical Studies. Antioxidants.

[26] Oudemans-van Straaten H.M., Man A.M.S., de Waard M.C. (2014). Vitamin C revisited. Crit. Care.

[27] Nowak D., Piasecka G., Antczak A., Pietras T. (1991). Effect of ascorbic acid on hydroxyl radical generation by chemical, enzymatic and cellular systems. Importance for antioxidant prevention of pulmonary emphysema. Biomed. Biochim. Acta.

[28] Buettner G.R., Jurkiewicz B.A. (1996). Catalytic Metals, Ascorbate and Free Radicals: Combinations to Avoid. Radiat. Res..

[29] Kaur B., Rowe B.H., Stovold E. (2013). Vitamin C supplementation for asthma. Cochrane Database Syst. Rev..

[30] Hartel C. (2004). Effects of vitamin C on intracytoplasmic cytokine production in human whole blood monocytes and lymphocytes. Cytokine.

[31] Bowie A.G., O’Neill L.A.J. (2000). Vitamin C Inhibits NF-κB Activation by TNF Via the Activation of p38 Mitogen-Activated Protein Kinase. J. Immunol..

[32] Carr A., Maggini S. (2017). Vitamin C and Immune Function. Nutrients.

[33] Spoelstra-de Man A.M.E., Elbers P.W.G., Oudemans-Van Straaten H.M. (2018). Vitamin C: Should we supplement?. Curr. Opin. Crit. Care.

[34] Dashti-Khavidaki S., Hajhossein Talasaz A., Tabeefar H., Hajimahmoodi M., Moghaddam G., Khalili H., Lessan-Pezeshki M., Jahanmardi A. (2011). Plasma Vitamin C Concentrations in Patients on Routine Hemodialysis and its Relationship to Patients Morbidity and Mortality. Int. J. Vitam. Nutr. Res..

[35] Papastephanidis C., Agroyannis B., Tzanatos-Exarchou H., Orthopoulos B., Koutsicos D., Frangos-Plemenos M., Kallitsis M., Yatzidis H. (1987). Re-evaluation of Ascorbic Acid Deficiency in Hemodialysed Patients. Int. J. Artif. Organs.

[36] Li X., Wang G., Chen D., Lu Y. (2014). Binding of ascorbic acid and α-tocopherol to bovine serum albumin: A comparative study. Mol. BioSyst..

[37] Canavese C., Petrarulo M., Massarenti P., Berutti S., Fenoglio R., Pauletto D., Lanfranco G., Bergamo D., Sandri L., Marangella M. (2005). Long-term, low-dose, intravenous vitamin C leads to plasma calcium oxalate supersaturation in hemodialysis patients. Am. J. Kidney Dis..

[38] Eiselt J., Racek J., Opatrný K., Trefil L., Stehlík P. (2006). The Effect of Intravenous Iron on Oxidative Stress in Hemodialysis Patients at Various Levels of Vitamin C. Blood Purif..

[39] Lim H.-S., Kim H.-S., Kim J.K., Park M., Choi S.J. (2019). Nutritional Status and Dietary Management According to Hemodialysis Duration. Clin. Nutr. Res..

[40] Panchal S., Schneider C., Malhotra K. (2018). Scurvy in a hemodialysis patient. Rare or ignored?. Hemodial. Int..

[41] Massicotte-Azarniouch D., McLean L., Brown P.A. (2019). Uremic leontiasis ossea due to secondary hyperparathyroidism complicated by vitamin C deficiency in a non-adherent chronic hemodialysis patient: A case report. Clin. Nephrol. Case Stud..

[42] Deicher R., Ziai F., Habicht A., Bieglmayer C., Schillinger M., Horl W.H. (2004). Vitamin C plasma level and response to erythropoietin in patients on maintenance haemodialysis. Nephrol. Dial. Transplant..

[43] Pincemail J., Vanbelle S., Degrune F., Cheramy-Bien J.-P., Charlier C., Chapelle J.-P., Giet D., Collette G., Albert A., Defraigne J.-O. (2011). Lifestyle Behaviours and Plasma Vitamin C and β-Carotene Levels from the ELAN Population (Liège, Belgium). J. Nutr. Metab..

[44] Hagel A.F., Albrecht H., Dauth W., Hagel W., Vitali F., Ganzleben I., Schultis H.W., Konturek P.C., Stein J., Neurath M.F. (2018). Plasma concentrations of ascorbic acid in a cross section of the German population. J. Int. Med. Res..

[45] Richter A., Kuhlmann M.K., Seibert E., Kotanko P., Levin N.W., Handelman G.J. (2008). Vitamin C deficiency and secondary hyperparathyroidism in chronic haemodialysis patients. Nephrol. Dial. Transplant..

[46] Zhang K., Liu L., Cheng X., Dong J., Geng Q., Zuo L. (2011). Low levels of vitamin C in dialysis patients is associated with decreased prealbumin and increased C-reactive protein. BMC Nephrol..

[47] Raimann J.G., Abbas S.R., Liu L., Larive B., Beck G., Kotanko P., Levin N.W., Handelman G. (2019). The effect of increased frequency of hemodialysis on vitamin C concentrations: An ancillary study of the randomized Frequent Hemodialysis Network (FHN) daily trial. BMC Nephrol..

[48] Coveney N., Polkinghorne K.R., Linehan L., Corradini A.M., Kerr P.G. (2011). Water-soluble vitamin levels in extended hours hemodialysis. Hemodial. Int..

[49] Rice M.E. (1999). Ascorbate compartmentalization in the CNS. Neurotox. Res..

[50] Tarng D.-C., Wei Y.-H., Huang T.-P., Kuo B.I.T., Yang W.-C. (1999). Intravenous ascorbic acid as an adjuvant therapy for recombinant erythropoietin in hemodialysis patients with hyperferritinemia. Kidney Int..

[51] Kennedy S.S., Perilloux A., Pereira R.C., Handelman G., Wesseling-Perry K., Salusky I.B. (2021). Vitamin C overload may contribute to systemic oxalosis in children receiving dialysis. Pediatr. Nephrol..

[52] (2000). Dietary Reference Intakes for Vitamin C, Vitamin E, Selenium, and Carotenoids.

[53] De Vriese A.S., Borrey D., Mahieu E., Claeys I., Stevens L., Vanhaeverbeke A., Roelens M., Langlois M.R. (2007). Oral Vitamin C Administration Increases Lipid Peroxidation in Hemodialysis Patients. Nephron Clin. Pract..

[54] Zhang K., Li Y., Cheng X., Liu L., Bai W., Guo W., Wu L., Zuo L. (2013). Cross-over study of influence of oral vitamin C supplementation on inflammatory status in maintenance hemodialysis patients. BMC Nephrol..

[55] Chan D., Irish A., Dogra G. (2005). Efficacy and safety of oral versus intravenous ascorbic acid for anaemia in haemodialysis patients. Nephrology.

[56] El Mashad G., ElSayed H., Nosair N. (2016). Effect of vitamin C supplementation on lipid profile, serum uric acid, and ascorbic acid in children on hemodialysis. Saudi J. Kidney Dis. Transplant..

[57] Yang C.-C., Hsu S.-P., Wu M.-S., Hsu S.-M., Chien C.-T. (2006). Effects of vitamin C infusion and vitamin E-coated membrane on hemodialysis-induced oxidative stress. Kidney Int..

[58] Ferretti G., Bacchetti T., Masciangelo S., Pallotta G. (2008). Lipid peroxidation in hemodialysis patients: Effect of vitamin C supplementation. Clin. Biochem..

[59] Zasowska-Nowak A., Nowak P.J., Bialasiewicz P., Prymont-Przyminska A., Zwolinska A., Sarniak A., Wlodarczyk A., Markowski J., Rutkowski K.P., Nowak D. (2016). Strawberries Added to the Usual Diet Suppress Fasting Plasma Paraoxonase Activity and Have a Weak Transient Decreasing Effect on Cholesterol Levels in Healthy Nonobese Subjects. J. Am. Coll. Nutr..

[60] Abdollahzad H., Eghtesadi S., Nourmohammadi I., Khadem-Ansari M., Nejad-Gashti H., Esmaillzadeh A. (2009). Effect of Vitamin C Supplementation on Oxidative Stress and Lipid Profiles in Hemodialysis Patients. Int. J. Vitam. Nutr. Res..

[61] Ramos R., Martínez-Castelao A. (2008). Lipoperoxidation and hemodialysis. Metabolism.

[62] Ruskovska T., Bennett S.J., Brown C.R., Dimitrov S., Kamcev N., Griffiths H.R. (2015). Ankyrin is the major oxidised protein in erythrocyte membranes from end-stage renal disease patients on chronic haemodialysis and oxidation is decreased by dialysis and vitamin C supplementation. Free Radic. Res..

[63] Conner T.A., McQuade C., Olp J., Pai A.B. (2012). Effect of intravenous vitamin C on cytokine activation and oxidative stress in end-stage renal disease patients receiving intravenous iron sucrose. BioMetals.

[64] Rodríguez-Ayala E., Anderstam B., Suliman M.E., Seeberger A., Heimbürger O., Lindholm B., Stenvinkel P. (2005). Enhanced RAGE-mediated NFκB stimulation in inflamed hemodialysis patients. Atherosclerosis.

[65] Samouilidou E., Grapsa E., Karpouza A., Lagouranis A. (2007). Reactive Oxygen Metabolites: A Link between Oxidative Stress and Inflammation in Patients on Hemodialysis. Blood Purif..

[66] Biniaz V., Sadeghi Shermeh M., Ebadi A., Tayebi A., Einollahi B. (2013). Effect of Vitamin C Supplementation on C-reactive Protein Levels in Patients Undergoing Hemodialysis: A Randomized, Double Blind, Placebo-Controlled Study. Nephrourol. Mon..

[67] Ivanovich P., Chenoweth D.E., Schmidt R., Klinkmann H., Boxer L.A., Jacob H.S., Hammerschmidt D.E. (1983). Symptoms and activation of granulocytes and complement with two dialysis membranes. Kidney Int..

[68] Baradari A.G., Zeydi A.E., Espahbodi F., Aarabi M. (2012). The effect of intravenous vitamin C on the phosphorus level reduction in hemodialysis patients: A double blind randomized clinical trial. Med. Glas..

[69] Płóciniczak A., Dzięgielewska-Gęsiak S., Brożek A., Blacha A., Nowicki M., Formanowicz D. (2018). High sensitivity C-reactive protein as a cardiovascular risk marker in independent community-living elderly persons. J. Biol. Regul. Homeost. Agents.

[70] Keypour H., Silver J., Wilson M.T., Hamed M.Y. (1986). Studies on the reactions of ferric iron with ascorbic acid. A study of solution chemistry using Mössbauer spectroscopy and stopped-flow techniques. Inorg. Chim. Acta.

[71] Duesterberg C.K., Cooper W.J., Waite T.D. (2005). Fenton-Mediated Oxidation in the Presence and Absence of Oxygen. Environ. Sci. Technol..

[72] Wiswedel I. (2009). F2-Isoprostanes: Sensitive Biomarkers of Oxidative Stress In Vitro and In Vivo: A Gas Chromatography-Mass Spectrometric Approach. Lipidomics.

[73] Marques de Mattos A., Afonso Jordão A., Abrão Cardeal da Costa J., Garcia Chiarello P. (2014). Study of Protein Oxidative Stress, Antioxidant Vitamins and Inflammation in Patients Undergoing either Hemodialysis or Peritoneal Dialysis. Int. J. Vitam. Nutr. Res..

[74] Nemet I., Monnier V.M. (2011). Vitamin C Degradation Products and Pathways in the Human Lens. J. Biol. Chem..

[75] Prasad A., Bekker P., Tsimikas S. (2012). Advanced Glycation End Products and Diabetic Cardiovascular Disease. Cardiol. Rev..

[76] Otero P., Viana M., Herrera E., Bonet B. (1997). Antioxidant and Prooxidant Effects of Ascorbic Acid, Dehydroascorbic Acid and Flavonoids on LDL Submitted to Different Degrees of Oxidation. Free Radic. Res..

[77] Kamgar M., Zaldivar F., Vaziri N.D., Pahl M.V. (2009). Antioxidant Therapy Does Not Ameliorate Oxidative Stress and Inflammation in Patients with End-Stage Renal Disease. J. Natl. Med. Assoc..

[78] Chan D., Irish A., Croft K.D., Dogra G. (2006). Effect of ascorbic acid supplementation on plasma isoprostanes in haemodialysis patients. Nephrol. Dial. Transplant..

[79] Cross J.M., Donald A.E., Nuttall S.L., Deanfield J.E., Woolfson R.G., Macallister R.J. (2003). Vitamin C improves resistance but not conduit artery endothelial function in patients with chronic renal failure. Kidney Int..

[80] Ashor A.W., Brown R., Keenan P.D., Willis N.D., Siervo M., Mathers J.C. (2019). Limited evidence for a beneficial effect of vitamin C supplementation on biomarkers of cardiovascular diseases: An umbrella review of systematic reviews and meta-analyses. Nutr. Res..

[81] Liakopoulos V., Roumeliotis S., Bozikas A., Eleftheriadis T., Dounousi E. (2019). Antioxidant Supplementation in Renal Replacement Therapy Patients: Is There Evidence?. Oxid. Med. Cell. Longev..

[82] Kelly Y.P., Weins A., Yeung M.Y. (2019). Accelerated Oxalosis Contributing to Delayed Graft Function after Renal Transplantation. Case Rep. Transplant..

[83] Palsson R., Chandraker A.K., Curhan G.C., Rennke H.G., McMahon G.M., Waikar S.S. (2020). The association of calcium oxalate deposition in kidney allografts with graft and patient survival. Nephrol. Dial. Transplant..

